# Modelling of Fractionated Condensation for Off-Flavours Reduction from Red Wine Fermentation Headspace

**DOI:** 10.3390/membranes12090875

**Published:** 2022-09-10

**Authors:** Maria João Pereira, António Ferreira, Carla Brazinha, João Crespo

**Affiliations:** 1 LAQV-REQUIMTE, Department of Chemistry, NOVA School of Science and Technology, FCT NOVA, Universidade NOVA de Lisboa, 2829-516 Caparica, Portugal; 2 iBET, Instituto de Biologia Experimental e Tecnológica, Apartado 12, 2780-901 Oeiras, Portugal

**Keywords:** aroma recovery, modelling fractionated condensation, red wine aroma, off-flavours, vapour permeation

## Abstract

A mathematical model of fractionated condensation is proposed for predicting the recovery and fractionation of target aromas from red wine fermentation headspaces in order to remove off-flavours. The applicability of the model is assessed for two different alternative processes: fractionated condensation and vapour permeation–fractionated condensation. The aromas of the headspace of red wine fermentation are commonly lost through the fermenter venting system and are enhanced by the stripping effect of the produced CO_2_. To mimic the operating conditions during the red wine fermentation, all experiments were performed at 30 °C with a red wine model solution containing relevant red wine aromas, the cosolvent ethanol at representative concentrations, and CO_2_. Both studied processes allow for a good recovery of esters in the 2nd condenser, with over 80% of ethyl acetate and isoamyl acetate recovery when using vapour permeation–fractionated condensation and a recovery of 84–96% of all esters when using fractionated condensation. However, only the integrated process of vapour permeation–fractionated condensation achieves a significant decrease in the amount of ethyl phenols (off-flavours compounds) in the 1st condenser, above 50%, as expected due to the use of an organophilic membrane. The developed model was validated experimentally for the integrated process, proving to be a highly valuable tool for the prediction of aroma fractionation, aiming at the removal of off-flavours.

## 1. Introduction

One of the most important wine quality indicators is its aroma profile, which comprises hundreds of distinct components that are responsible for the wine’s flavour and odour [1,2]. The food flavour industry is expected to be worth 20.12 billion US dollars by 2028 [3]. Several alcoholic beverages are available on the market, including wine, beer, cider, and spirits, with the world’s top players accounting for more than 60% of global sales [4].

The presence of off-flavours in an alcoholic beverage may lead to a consumer’s perception of inferior quality, which can be extremely costly to the industry [5]. Volatile phenols, particularly 4-ethyl phenol and 4-ethyl guaiacol, are aroma molecules that, at the perception threshold limit, compromise wine quality by imparting aroma defects such as “horse sweat,” “animal,” “leather,” and “medicinal” [1]. According to the literature, preventing the generation of volatile phenols by *Brettanomyces* is the major problem in contemporary winemaking and is to blame for considerable economic losses globally [6,7].

The synthesis of these compounds, which often happens during fermentation by *Brettanomyces/Dekkera bruxellensis*, results in the wine characteristic known popularly as “Brett-taint” [8]. Off-flavour defects caused by the presence of these molecules are one of the most common organoleptic issues encountered during the production of many fermented alcoholic drinks (e.g., wine, beer, cider, etc.) [5].

The preservation of wine aroma during processing is also a critical topic in food technology that is becoming increasingly important. The demand for high-quality and widely characterised products to compete in the global market through differentiation has become imperative in many industries [9]. The amount of volatile compounds produced at the end of the fermentation process is mostly controlled by yeast synthesis, while it is also influenced by depletion due to the CO_2_ stripping effect, which drags aromas to the fermenter headspace, where they are lost by venting. During wine fermentation, huge amounts of CO_2_ (up to 40 L/L of must) are rapidly emitted, resulting in a continuous stripping-off of volatile compounds, with up to 70% of the produced volatile compounds being stripped away [10,11]. To minimise the resultant deterioration of the final aroma bouquet, these aroma compounds should be recovered and reintroduced into the final product [12].

Condensation can be performed at different temperatures in a sequence of condensation steps, aiming to achieve fractionated condensation and separate fractions enriched with target compounds. The temperature of each condenser must be controlled in accordance with the downstream pressure and its separation and recovery characteristics [13]. The various components are condensed differently, according to operating conditions and the liquid–vapour equilibrium [14].

Mathematical models of fractionated condensation were previously studied when this operation was integrated with pervaporation. Pervaporation-fractionated condensation processes from wine model solutions were investigated by Brazinha et al. [15]. A mathematical model was developed for the fractionated condensation step to evaluate the influence of the noncondensable gas CO_2_ and the cosolvent ethanol on the recovery of aroma condensates from aqueous and hydroalcoholic solutions. Pereira et al. [16] studied pervaporation-fractionated condensation from by-products of the seafood industry to remove their off-flavours. These mathematical models of fractionated condensation require information about feed composition, are based on mass balances, and assume thermodynamic equilibrium. If this assumption is not valid, the model cannot predict the composition of each condenser. In this work, the fractionated condensation model is extended to two different processes—a stand-alone fractionated condensation and an integrated vapour permeation/fractionated condensation—aiming at the recovery of red wine aromas free from off-flavours.

## 2. Experimental Section

### 2.1. Materials

In the vapour permeation experiments, the hydrophobic dense membrane PervapTM 4060 (DeltaMem AG, Allschwil, Switzerland) was used. It is a commercial flat sheet membrane with a polydimethylsiloxane (PDMS) active top layer with a thickness of 2 μm and an effective membrane area of 10^−2^ m^2^. The solvents used in the model solutions were mineral carbonated water (brand Vimeiro, Águas do Vimeiro S.A., Maceira, Portugal) and ethanol (Panreac, Barcelona, Spain, 99.5% purity). The five aromas chosen as model components were ethyl acetate (Sigma–Aldrich, St. Louis, MO, USA, ≥99% purity), ethyl hexanoate (Sigma–Aldrich, St. Louis, MO, USA, ≥99% purity), isoamyl acetate (Sigma–Aldrich, St. Louis, MO, USA, ≥97% purity), 4-ethyl phenol (Sigma–Aldrich, St. Louis, MO, USA, ≥99% purity), and 4-ethyl guaiacol (Sigma–Aldrich, St. Louis, MO, USA, ≥98% purity). A DVB/CAR/PDMS fibre, 50/30 μm, 2 cm, and a Sapiens, Wax-MS (TeknoKroma, Barcelona, Spain) were used for SPME/GC-MS analysis.

#### 2.1.1. Definition of the Model Solution of the Red Wine Fermentation Headspace

A model solution was defined, mimicking a red wine fermentation headspace, prepared with mineral carbonated water (brand Vimeiro, Águas do Vimeiro S.A., Maceira Portugal), which was further supersaturated with carbon dioxide (Air Liquide, Paris, France, 99.95% purity) administrated at a flow rate of 100 L of CO_2_ L^−1^.h^−1^, and ethanol (Panreac, Barcelona, Spain, 99.5% purity) was added at 10% *wt* in water. 

The aroma compounds were chosen considering the most common esters present in red wines and the off-flavours generated during the vinification process. 

The properties of each chosen aroma of the model solution are listed in Table 1A. The thermodynamic parameters and organoleptic qualities are summarised in Table 1B, which includes activity coefficients at infinite dilution and the saturation vapour pressure.

The activity coefficient values presented for aqueous solutions were found in the literature [15,19]. The influence of ethanol on the aroma activity coefficient is well discussed in the literature, resulting in a decrease in these values [15,20]. The Pierotti modified parameters obtained by Equation (1) were used to calculate the activity coefficients of the aromas in a mixture with 10% ethanol:

(1) $$\gamma_{25\;{}^{\circ}C,10\%\;Etoh\;}^{\infty }=\gamma_{25\;{}^{\circ}C,water}^{\infty }\cdot \left[\frac{\gamma_{70\;{}^{\circ}C,10\%\;Etoh}^{\infty }}{\gamma_{70\;{}^{\circ}C,water}^{\infty }}\right]$$

where the activity coefficients at 25 °C in water and at 70 °C, both in water and in a 10% ethanol aqueous solution, were determined in [19].

### 2.2. Experimental Setup

#### 2.2.1. Fractionated Condensation with CO_2_ Stripping Gas Experiments 

**Figure 1:**
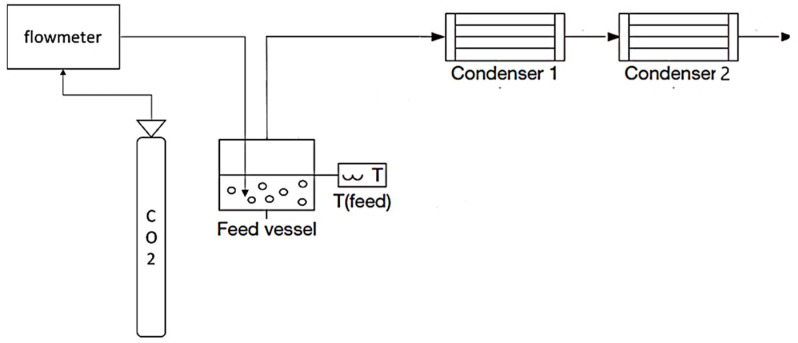
Diagram of the experimental setup of fractionated condensation with CO_2_ stripping gas with two condensers connected in series.

The experimental setup, presented in Figure 1, was built to recover aromas by CO_2_ stripping without the assistance of a membrane, followed by fractionated condensation. This setup consists of a feed vessel with CO_2_ supersaturation by the injection of gas inside the model solution, which aims to mimic the wine fermentation vessel. The vapour stream was produced in the same way as in the integrated vapour permeation–fractionated condensation. All the tubing was stainless steel, and the fractionated condensation approach included two U-shaped glass trap condensers in series. The first condenser was refrigerated using an FP 500-MC (Julabo, Seelbach, Germany), and liquid nitrogen was used to submerge the second condenser.

#### 2.2.2. Vapour Permeation with CO_2_ Stripping Gas and Fractionated Condensation 

**Figure 2:**
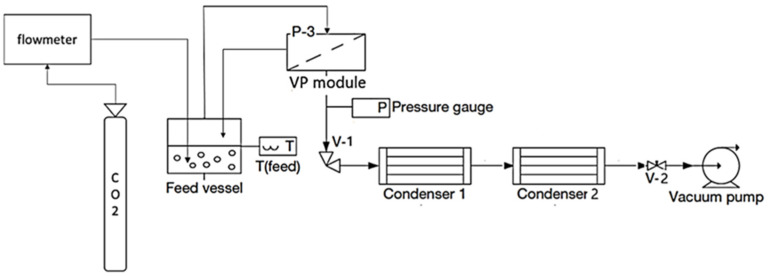
Diagram of the experimental setup of a vapour permeation apparatus with two condensers connected in series.

Figure 2 illustrates the experimental setup used, which was planned to recover aromas using vapour permeation with CO_2_ stripping gas. A CO_2_ bottle was linked to a flowmeter, and CO_2_ was injected into the model solution. Working at a constant CO_2_ flow rate of 100 L of CO_2_ L^−1^·h^−1^ and a feed temperature of 30 °C, a vapour stream was generated, emulating the vapour stream generated in a red wine fermenter. A radial flat module (GKSS, Geesthacht, Germany) was tested, which was described in detail in [19]. The upstream tubing was either Viton or Teflon, and the metal used in the unit was stainless steel. In this study, a E2M5 rotary vane dual-stage mechanical vacuum pump (Edwards, Burgess Hill, UK) ensured vacuum conditions on the permeate side, with a *p_perm_* of 1000 Pa. The apparatus was equipped with a TPR280 pressure gauge (Pfeiffer Vacuum, Aßlar, Germany), which collected permeate pressure measurements of *p_perm_* (Pa). The downstream pressure, *p_perm_* (Pa), was regulated by an RVC 300 pressure controller (Pfeiffer Vacuum, Aßlar, Germany), which varied the resistance produced by an RME 005 electro valve (V2) (Pfeiffer Vacuum, Germany). The downstream circuit incorporated two condensation U-shape glass trap condensers in series, using an FP 500-MC (Julabo, Seelbach, Germany) to refrigerate the first condenser. Liquid nitrogen was used to submerge the second condenser.

### 2.3. Operating Conditions

#### 2.3.1. Feed Compartment

**Figure 3:**
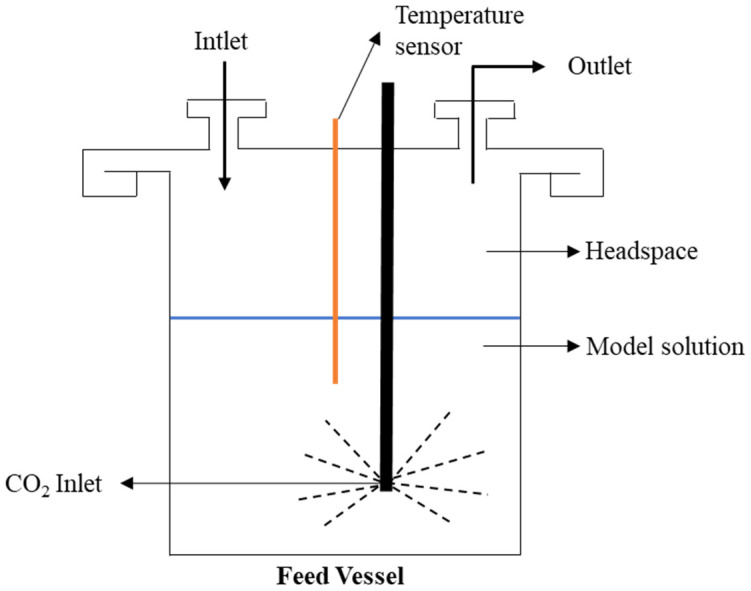
Scheme of the feed vessel.

A volume of 3 L of the model solution with aromas of red wine (see Section 2.1.1) was placed in the vessel with a headspace of 2 L (See Figure 3). The runtime of each trial was established at 3 h, based on the ratio between the membrane area of 10^−2^ m^2^ and the volume of the feed vessel. Considering the limited experimental time, previous membrane conditioning was performed by permeating ethanol at 10% *wt* in mineral carbonated water for 4 h before each vapour permeation experiment. The temperature of the feed vessel was kept constant at 30 °C throughout all experiments.

#### 2.3.2. Fractionated Condensation Experiments 

In the fractionated condensation experiments, the following parameters were controlled: the temperature of the feed stream *T_feed_* was maintained at 30 ± 1 °C, and the CO_2_ flow rate was kept constant at 100 L of CO_2_ L^−1^ h^−1^. The temperature of the first condenser, *T_1,condens_*, was set at −40 °C, and the temperature of the second condenser, *T_2,condens_*, was −196 °C. The experiments were performed at atmospheric pressure.

#### 2.3.3. Vapour Permeation–Fractionated Condensation Experiments 

In the vapour permeation experiments, different parameters were controlled: the temperature of the feed stream *T_feed_* was maintained at 30 ± 1 °C, and the CO_2_ flow rate was kept constant at 100 L of CO_2_ L^−1^ h^−1^. Despite being higher than the CO_2_ production rate of wine fermentation, this flow rate was selected to ensure a gas stripping effect on the aromas present in the model solution and to obtain a CO_2_-supersaturated feed with an upstream pressure above atmospheric pressure. The temperature of the first condenser, *T_1,condens_,* was studied at −40, −25, and −15 °C, while the temperature of the second condenser, *T_2,condens_,* was set at −196 °C. The permeate pressure, *p_perm_,* of 1000.0 ± 50.0 Pa was chosen to ensure a good trade-off between energy consumption and transport driving force (Brazinha et al. [15]). A downstream pressure of 1 kPa is commonly reported for pervaporation industrial processes.

### 2.4. Analytical Methods

Analyses by SPME-GC-MS were carried out using an AOC-5000 Plus autosampler (Shimadzu, Kyoto, Japan) and a GC-MS-QP2000 (Shimadzu, Kyoto, Japan). A DVB/CAR/PDMS fibre, 50/30 μm, 2 cm, and a Sapiens Wax-MS (TeknoKroma, Barcelona, Spain) chromatographic column with 60 m × 0.25 mm i.d. × 0.25 μm was used. The fibre was subjected to a temperature of 40 °C for 15 min, with 250 rpm agitation in the head space inside the hermetic vial containing 7 mL of the sample. After this, the compounds were desorbed in the injector at 250 °C for 10 min with a 1:10 split ratio. Helium, at 4 mL/min, was applied as a carrier gas, and the chromatographic programme was started at a temperature of 40 °C and kept for 5 min, increased by 5 °C/min up to 170 °C, increased by 30 °C/min up to 230 °C, and maintained for 4 min. The temperatures of the ionisation source and the detector were 245 °C and 250 °C, respectively. Detection was performed in the m/z 29–300 range. The analysis were performed in triplicate.

### 2.5. Modelling of Fractionated Condensation Step

A mathematical model is necessary to simulate the capture of each aroma in the two in-series condensers to achieve successful fractionated condensation and remove off-flavours from potentially important target aromas. Such a model should also allow users to identify the best operating temperatures in each condenser to separate off-flavours from desirable aromas. Supported by the work carried out by Brazinha et al. [15], a simple mathematical model for designing a fractionated condensation system, comprised of a set of condensers, can be established to achieve off-flavour removal [16]. 

**Figure 4:**
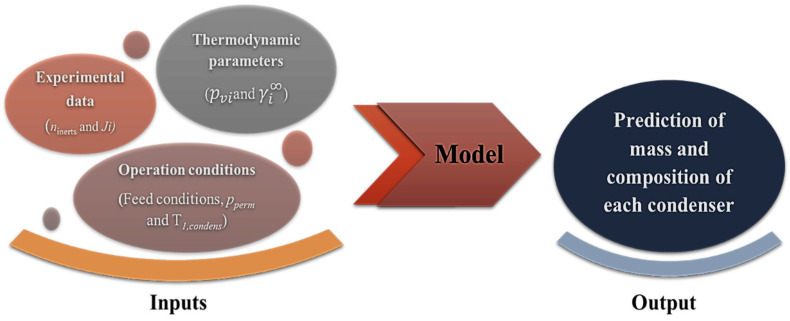
Diagram representing the flux of data in this work.

The model is built on the assumption that the vapour stream in a series of condensers (non-total condensation) is in thermodynamic equilibrium with the liquid condensate phase in each condenser. This model uses the feed composition and feed flow operating parameters as well as the temperature of the first condenser as input variables (see Figure 4). The second condenser was assumed to achieve total condensation due to the low temperature of operation. The model simulation predicts the mass and composition of the condensate in each condenser. It is possible to simulate the composition of the condensates obtained in the condensers by starting with inputs such as: (i) the permeate flux of each aroma under study, (ii) thermodynamic parameters (saturation vapour pressure and activity coefficient at infinite dilution), and (iii) the operating conditions of pressure and temperature applied in upstream and downstream compartments. This is a very effective model for determining the optimal operating parameters for achieving the desired separation of valuable aromas from off-flavours.

For aqueous systems, the model was extensively explained in a previous paper [16]. Through a system of equations that constitute the model, we were able to select the best operating conditions to achieve the best separation of valuable flavours from off-flavours. Equations (2) and (3) allow the determination of the percentage of water and aroma(s) condensation of in the first condenser, respectively:

(2) $$\%condens_{w1}=1-\frac{n_{inert}}{n_{w0}}\cdot \frac{p_{vw\left(T1,\;condens\right)}}{p_{perm}-p_{vw\left(T1,\;condens\right)}}$$

(3) $$\%condens_{aroma1}≅1-\frac{n_{inert}}{n_{aroma0}}\cdot \frac{\varkappa_{aroma1}\cdot \gamma_{aroma1}^{\infty }\cdot p_{varoma\left(T1,\;condens\right)}}{p_{perm}-p_{vw\left(T1,\;condens\right)}}$$

where *n_inert_* is the inert gas (CO_2_) molar flow rate in the stream, *P_vw_* is the saturation vapour pressure of water or aroma, *p_perm_* is the permeate pressure applied to the system, *n_w_ _or aroma0_* is the molar flow rate of each species before the first condenser, *ϰ_w_ _or aroma_* is the molar fraction in the feed, and *ϒ*^∞^*_aroma_* is the activity coefficient at infinite dilution of the aroma(s).

It is important to highlight that this model can handle multicomponent aroma systems because the target aroma compounds are extremely diluted in the feed solutions in most real-case scenarios. Therefore, each aroma behaves independently under these highly diluted aroma concentration conditions, with no flow coupling between them [21]. Furthermore, diluted aroma compounds have no effect on water and ethanol transport [22].

This model was applied and experimentally validated for aqueous systems [16]. Following the strategy established for aqueous solutions, the model was developed for more complex solutions comprising ethanol and the presence of noncondensable gases.

(4) $$x_{w1}+x_{et1}=1$$

(5) $$x_{w2}=\frac{n_{w1^{\prime }}}{n_{w1^{\prime }}+n_{et1^{\prime }}}$$

(6) $$n_{w0}+n_{et0}=n_{T1}+n_{T2}$$

(7) $$n_{w0}≅x_{w1}\cdot n_{T1}+x_{w2}\cdot n_{T2}$$

(8) $$\%condens_{i1}=1-\frac{n_{inerts}}{n_{i0}}\cdot \frac{x_{i1}\cdot \gamma_{i1}\cdot p_{vi}\left(T_{1,condens}\right)}{p_{perm}-x_{w1}\cdot \gamma_{w1}\cdot p_{vw}\left(T_{1,condens}\right)-x_{et1}\cdot \gamma_{et1}\cdot p_{vet}\left(T_{1,condens}\right)}\;i\;=\;w,\;et,\;aroma$$

With Equations (2) and (3), it is possible to calculate the percentage of water and aroma(s) obtained in the first condenser. Due to the very diluted concentration of the aromas present in the stream, it was assumed that they did not affect the water and ethanol condensation. In Equation (5), it is considered a fact that the second condenser was used as a total condenser at a temperature of −196 °C. Considering this set of equations, a modified expression for the determination of the percentage of water, ethanol, and aroma(s) condensation in the first condenser is obtained in Equation (8).

The Antoine law equation was used to estimate the saturation vapour pressure of water, ethanol, and other aromas at the temperature range using the Antoine law constants shown in Table 2.

For the estimation of the inert gases’ molar flow, *n_inert_* (mol/s), at the end of the vapour permeation trials, the vacuum pump was closed and the rising downstream pressure, *p_perm_* (Pa), was monitored over time. The molar flow rate of inert gases is calculated by the ideal gas equation using the slope of the function Δ*p_perm_*/Δt (Pa/s) above the saturation vapour pressure of water. At 30 °C, the obtained value of *n_inert_* was 9.24 × 10^−6^ ± 2.00 × 10^−8^ mol·s^−1^. In the evaporation experiments at atmospheric pressure, the experimentally determined inert flux was 3.72 × 10^−3^, equivalent to the CO_2_ constant flow rate used.

## 3. Results and Discussion

### 3.1. Fractionated Condensation of the Model Solution of Red Wine

A model red wine solution was used under defined upstream operating conditions to originate a vapour that mimics the headspace of wine fermentation. The aromas were found in a range of concentrations in the feed vessel in the model solution (0.6–50.0 ppm). The vapour formed inside the feed vessel was the starting point of the model developed in this work. To understand how effective the removal of off-flavours can be, modelling of the fractionated condensation process at atmospheric pressure was performed.

**Figure 5:**
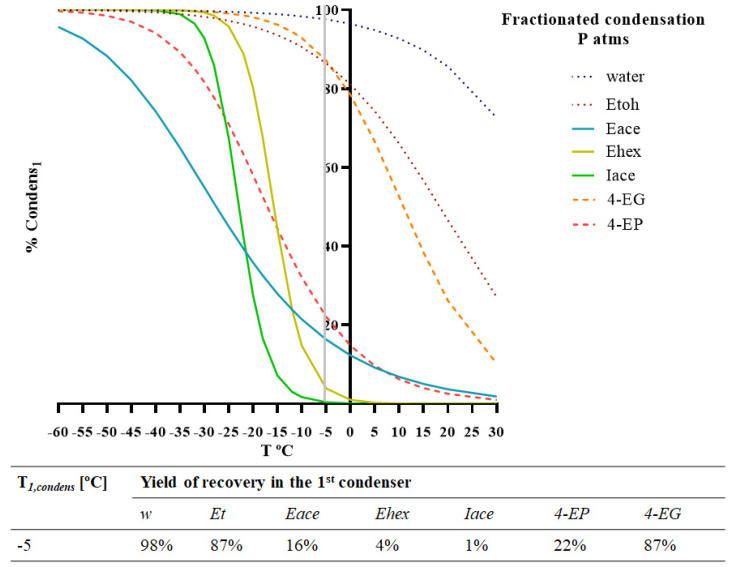
Model simulation of fractionated condensation for two different chemical families (esters and ethyl phenols) included in the red wine model solution. Percentage of condensation of each compound present (water, ethanol, ethyl acetate, ethyl hexanoate, isoamyl acetate, 4-ethyl phenol, and 4-ethyl guaiacol) in the 1st condenser (*%Condens_i1_*). Operating conditions: *T_feed_* = 30 °C; *p_perm_* = 101 kPa.

To achieve a reduction in the ethyl phenol content from the desirable aroma concentrate, the aroma fractionation aimed at obtaining two different condensates, where an optimal condensation temperature (in the first condenser) allowed the separation of valuable aromas from off-flavours. The mathematical model was used to simulate the percentage of each compound, *i,* recovered in the first condenser, *%Condens_i1_*, for different values of *T_1,condens_* (°C]), which are presented in Figure 5.

The optimal temperature suggested by the simulation was −5 °C. With this condition, the model estimated a recovery of more than 87% of 4-EG, 22% of 4-EP, 16% of ethyl acetate, and a residual (*%Condens_i1_* < 4%) of ethyl hexanoate and isoamyl acetate. These results represent a good recovery of the target esters in the 2nd condenser and a good reduction in the ethyl phenol content, more expressive in the case of 4-EG. 

Due to the lower energy costs involved, fractionated condensation allows for aroma recovery under more economical conditions. However, this process does not allow for proper off-flavour fractionation, leaving 78% of 4-EP, the most important off-flavour, in the 2nd condenser.

### 3.2. Vapour Permeation–Fractionated Condensation Processing of the Model Solution of Red Wine

The application of vapour permeation in the industry has been explored since the early 1980s, and it is now widely applied in the petrochemical and chemical sectors for the manufacture and purification of volatile organic compounds [25]. For this reason, it was considered relevant to test this model in an integrated vapour permeation/fractionated condensation process.

**Figure 6:**
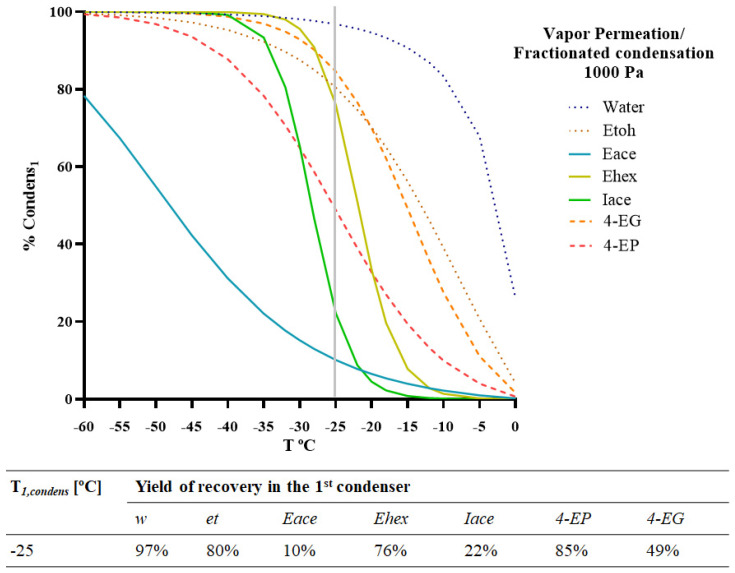
Model simulation of the integrated system of vapour permeation/fractionated condensation for two different chemical families (esters and ethyl phenols) included in the red wine model solution. Percentage of condensation of each compound present (water, ethanol, ethyl acetate, ethyl hexanoate, isoamyl acetate, 4-ethyl phenol, and 4-ethyl guaiacol) in the 1st condenser (*%Condens_i1_*). Operating conditions: Pervap 4060 membrane; *Tfeed* = 30 °C; *p_perm_* = 1000 Pa.

To find the best *T_1,condens_* (°C) to achieve the removal of the ethyl phenols for vapour permeation/fractionated condensation at a permeate pressure of 1000 Pa, a simulation was performed on the fractionated condensation process, and the *%Condens_i,1_* was predicted for different values of *T_1,condens_* (°C). The obtained results are presented in Figure 6.

Based on the simulation results, a temperature range of −25 to −15 °C may be suggested. The simulation showed that at a *T_1,condens_* (°C) of −15 °C, the 1st condenser ensures a residual condensation (*%Condens_i_*_1_ < 8%) of all the esters present in the model solution as well as condensation of 20 and 50% of 4-EP and 4-EG, respectively. The model predicted a retention of more than 80% for 4-EG, 75% for ethyl hexanoate, around 50% for 4-EP, and less than 20% for ethyl acetate and isoamyl acetate at a *T_1,condens_* (°C) of −25 °C. This represents a good recovery of some esters in the 2nd condenser and a good reduction in the ethyl phenol content. However, it implies the loss of a substantial part of the ethyl hexanoate content. The obtained results show that a complete separation of volatile phenols from the target esters is not possible by the proposed integrated process. However, it allows for a good reduction in the ethyl phenols.

Other research studies of fractionated condensation of wine aromas have shown similar behaviour concerning the condensation of esters. Brazinha et al. [15], aiming at the recovery and/or fractionation of aromas, studied the performance of vacuum-fractionated condensation integrated with an organophilic pervaporation process. Using a *T_1,condens_* (°C) of −9 °C, a similar *%Condens_i1_* of 9% for ethyl acetate was obtained. In addition, Ribeiro et al. [12] studied aroma extraction by gas stripping in a bubble column followed by vapour permeation and compared it with the pervaporation process. By applying a multistage condensation system in series and a *T_1,condens_* (°C) of −30 °C, it was found that ethyl acetate remained in the vapour phase, leading to the recovery of pure water. The ester recovery was carried out in a second cold trap set to −117 °C. Regarding ethyl phenol removal, the only integrated system using membrane processes reported in the literature was a reverse-osmosis operation followed by a hydrophobic adsorptive resin, which showed significant reductions in 4-ethylphenol and 4-ethylguaiacol concentrations. However, there was a loss of other aroma compounds [1].

### 3.3. Model Validation with Experimental Values

**Figure 7:**
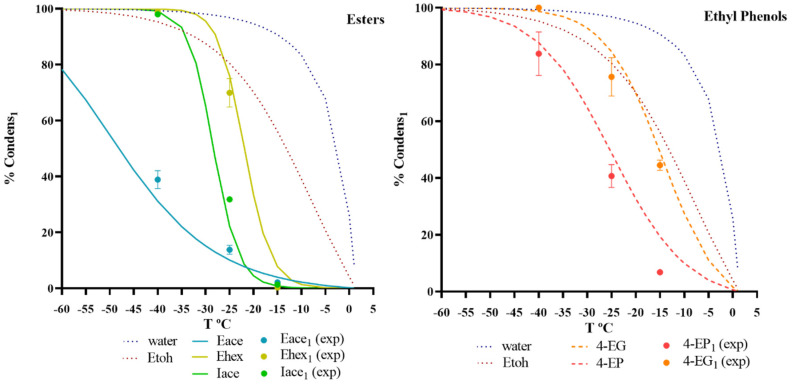
Validation of the model simulation for the different chemical families (esters and ethyl phenols) included in the red wine model solution. Percentage of condensation of each compound present (water, ethanol, ethyl acetate, ethyl hexanoate, isoamyl acetate, 4-ethyl phenol, and 4-ethyl guaiacol) in the 1st condenser (*%Condens_i1_*). Operating conditions: Pervap 4060 membrane; *T_feed_* = 30 °C; *p_perm_* = 1000 Pa; The symbols represent the experimental values, whereas the lines show simulated data.

The values of *%Condens_i1_* related to vapour permeation–fractionated condensation were simulated based on the model explained in the modelling of fractionated condensation step (Section 2.5. using the input parameters: *ϰ_i,feed_*, *T_feed_*, *p_perm_*, *Ji*, and *n_inert_* and varying *T_1,condens_*. The red wine headspace model solution composition, including esters and ethyl phenols, is presented in Table 1A,B (Section 2.1.1). Figure 7 illustrates the predicted and experimental percentages of recovery for each compound, *i,* in the first condenser. A good agreement between the experimental and simulated results of *%Condens_i1_* was obtained. As described in Section 3.2, applying a *T_1,condens_* (°C) of −25 °C results in a reduction in the off-flavour concentration with the fractionated condensation.

Even in the presence of noncondensable gases, this model offers a simple and efficient approach for the simulation of the percentage of condensation of each compound in each condenser at a given downstream pressure and condenser temperature. For some of the aromas studied, the values of *%Condens_i1_* were slightly overestimated, which was explained by a lower condensation efficiency due to the inert gas stripping effect, which could not be predicted thermodynamically.

## 4. Conclusions

Fractionated condensation, as a stand-alone step or in an integrated vapour permeation–fractionated condensation system, exhibited a good recovery of esters. However, only the vapour permeation–fractionated condensation system allowed for obtaining a significant decrease in the amount of ethyl phenols, with a retention in the 1st condenser of over 80% for 4-EG and over 50% for 4-EP.

Even considering a complex feed solution, as in the case of a red wine fermentation headspace with ethanol and CO_2_, this model proved that it might be applied as a quantitative tool to assess whether separation between a target valuable aroma and an off-flavour is feasible and which conditions are most appropriate to achieve an optimal separation with minimal experimental work.

The modelling strategy outlined and validated in this study is a straightforward tool that can be applied to a variety of integrated processes. It requires little experimental data and enables process operating conditions to be optimised in order to obtain a desired condensate composition.

## Figures and Tables

**Table 1:** Composition of the model solution of red wine: (**A**) characterisation of the selected red wine aroma compounds and (**B**) the physicochemical properties of the aroma compounds.

**(A)**	**Aroma** **Compound**	**Family of Chemicals**	**Reason to Include the Aroma in the Model** **Solution**			**Concentration of the Model Solution**
	Ethyl acetate	Esters	Esters are the most abundant aromas produced by wine yeasts. Ethyl acetate, isoamyl acetate and ethyl hexanoate are considered the main component of a fruit flavour [17]			50 ppm
	Ethyl hexanoate					8 ppm
	Isoamyl acetate					6 ppm
	4-ethylphenol	Phenols	When volatile phenols reach limiting concentrations *, they affect the quality of the wine leading to aroma defects normally described as “horse sweat”, “animal” and “medicinal” [6]			0.6 ppm
	4-ethylguaiacol					1 ppm
	* The odour threshold is 440 ppb for 4-EP and 33 ppb for 4-EG [18].					
**(B)**	**Compound**	**MW (g/mol)**	**BP (°C)**	**P^vi^_25 °C_ (Pa)**	***ϒ* ^∞^_25 °C, water_**	***ϒ* ^∞^_25 °C, 10% Etoh_**
	Ethyl acetate	88.11	77.10	12,622.12	50	37
	Ethyl hexanoate	144.21	167.00	49,898.73	12,615	9014
	Isoamyl acetate	130.18	142.50	1,470,959.90	3865	2280
	4-ethyl guaiacol	152.19	236.50	7.56	8383	*
	4-ethyl phenol	122.16	217.90	33.19	23,742	*
	(MW = molecular weight, BP = boiling point, pvi = saturation vapour pressure, *ϒ*∞ = activity coefficient at infinite dilution). * For ethyl phenols, the activity coefficient of the aromas in a mixture with 10% ethanol was not calculated due to the lack of information about the activity coefficients at 70 °C in water					

**Table 2:** Antoine law constants for each compound studied.

Compound	A	B	C	Range of Temperature (°C)	Reference
Water	5.40	1838.68	−31.74	−015 to 29.85	[23]
Ethanol	5.25	1598.67	−46.42	−0.15 to 78.55	[23]
Ethyl acetate	4.23	1245.70	−55.19	-	[23]
Ethyl hexanoate	15.99	3118.28	−106.76	-	[19]
Isoamyl acetate	16.50	2871.68	−110.92	-	[19]
4-Ethyl phenol	7.62	1955.30	195.46	99.76 to 244.80	[23]
4-Ethyl guaiacol	7.90	2203.80	234.22	85.27 to 233.09	[24]

## Data Availability

The data presented in this study are available on request from the corresponding author.

## Nomenclature

Variables and notations	
*%Condens*	percentage of condensation (%)
*A*	first constant of the Antoine law (−)
*B*	second constant of the Antoine law (°C for the ethyl phenols and K for the other compounds)
*C*	third constant of the Antoine law (°C for the ethyl phenols and K for the other compounds)
*Hi*	Henry’s law constant of compound (−)
*n*	molar flow rate (mol·s^−1^)
*p_i_*	partial pressure of compound *i* (Pa)
*p_vi_*	saturation vapour pressure of pure compound *i* (Pa)
*p_perm_*	total permeate pressure (Pa or mbar)
*t*	time (s)
*T*	temperature (K or °C)
*V*	volume (mL)
*x*	liquid molar fraction (−)
*y*	gas phase molar fraction (−)
*α*	selectivity (−)
*ϒ* ^∞^ *_i_*	activity coefficient of compound *i* in infinite dilute aqueous solution (−)
Subscripts (compounds)	
*w*	water
*et*	ethanol
*i*	compound *i*
*j*	compound *j*
*inerts*	inert gases
In relation to the membrane	
*feed*	in the feed side
*perm*	in the permeate side
Streams	
*0*	between the pervaporation module and the first condenser
*1*	in the first condenser
*1’*	between condensers
*2*	in the second condenser
*condens*	condenser
